# The Circular Stapled Esophagogastric Anastomosis in Esophagectomy: No Differences in Anastomotic Insufficiency and Stricture Rates Between the 25 mm and 28 mm Circular Stapler

**DOI:** 10.1007/s11605-020-04895-x

**Published:** 2021-01-27

**Authors:** E. Tagkalos, P. C. van der Sluis, E. Uzun, F. Berlth, J. Staubitz, I. Gockel, R. van Hillegersberg, H. Lang, Peter P. Grimminger

**Affiliations:** 1 Department of General, Visceral and Transplant Surgery, University Medical Center of the Johannes Gutenberg University Mainz, Mainz, Germany; 2 Department of Visceral, Transplant, Thoracic and Vascular Surgery, University Hospital of Leipzig, Leipzig, Germany; 3 Department of Surgery, University Medical Center Utrecht, Utrecht, The Netherlands

**Keywords:** Ivor-Lewis Esophagectomy, Circular stapled anastomosis, Esophago-gastric anastomosis

## Abstract

**Background:**

For patients undergoing an Ivor Lewis esophagectomy with a circular stapled anastomosis, the optimal diameter of the used circular stapler to restore continuity is unknown. The aim of this study was to compare the 25 mm stapled versus the 28 mm stapled esophagogastric anastomosis after Ivor Lewis esophagectomy, focusing on anastomotic insufficiency and postoperative anastomotic strictures.

**Methods:**

Between February 2008 and June 2019, 349 consecutive patients underwent Ivor Lewis esophagectomy with gastric conduit reconstruction and circular stapled anastomosis. Patient characteristics and postoperative results, such as anastomotic insufficiency rates, postoperative anastomotic stricture rates, time to anastomotic stricture rate, and the number of dilatations, were recorded in a prospective database and analyzed.

**Results:**

In 222 patients (64%), the 25 mm circular stapler was used and in 127 patients (36%) the 28 mm circular stapler was used. There were no differences in baseline characteristics. Anastomotic insufficiency rates were comparable between the 25 mm (12%) and the 28 mm groups (11%) (*p* = 0.751). There were no differences between postoperative anastomotic strictures in the 25 mm (14%) and the 28 mm groups (14%) (*p* = 0.863). Within patients with postoperative anastomotic strictures, a median number of 2 dilatations were observed in each group (*p* = 0.573) without differences in the time to first diagnosis (*p* = 0.412).

**Conclusion:**

There were no differences in anastomotic insufficiency and postoperative anastomotic stricture rates between the 25 mm and the 28 mm circular stapled esophagogastric anastomosis after Ivor Lewis esophagectomy. Both the 25 mm and 28 mm stapler can be safely used to create a circular stapled esophagogastric anastomosis to restore continuity after esophagectomy.

## Background

Neoadjuvant treatment followed by esophagectomy with 2-field lymphadenectomy is the golden standard in the surgical treatment of patients with esophageal cancer.^1^–^3^ Compared to an open transthoracic esophagectomy (robot assisted), minimally invasive esophagectomy results in a lower percentage of postoperative complications.^4^,^5^ After esophagectomy, continuity of the gastrointestinal tract is restored by creating a gastric conduit with an esophagogastric anastomosis. However, it is currently unknown, if a McKeown procedure (cervical anastomosis) or an Ivor Lewis procedure (intrathoracic anastomosis) should be preferred for patients with esophageal cancer undergoing esophagectomy.^6^,^7^ In our hospital, the Ivor Lewis procedure is the preferred therapeutic option for patients undergoing esophagectomy, based on the hypothesis that high-intrathoracic anastomosis is to be associated with a lower anastomotic insufficiency rates, lower rates of recurrent laryngeal nerve palsy, and a shorter in-hospital stay, as compared to cervical anastomosis.^8^

Different surgical techniques exist to create the (minimally invasive) intrathoracic esophagogastric anastomosis in the context of Ivor Lewis procedure: hand sewn, linear stapled, and circular stapled.^9^–^12^ Few studies have suggested superiority of a stapled over a hand-sewn anastomosis considering insufficiency rates.^13^,^14^ Therefore, the circular stapled esophagogastric anastomosis is the preferred surgical technique to restore upper gastrointestinal continuity in our hospital.^12^

For a circular stapled anastomosis, different stapler diameters are available, and the most frequently used ones have a diameter of 25 mm and 28 mm. In a recent meta-analysis, different circular stapler diameters used for the esophagogastric anastomosis were compared.^13^ It was shown that the use of a 28 mm circular stapler was strongly associated with a reduced risk of postoperative anastomotic stricture in the upper GI tract compared to a 25 mm circular stapler.^14^

In this article, we present our experience with the 25 mm and 28 mm circular stapler devices for esophagogastric anastomosis after esophagectomy in a large volume tertiary referral center for esophageal cancer. The aim of this study was to compare the incidence of anastomotic insufficiency and anastomotic strictures associated with the two different sizes.

## Methods

Between February 2008 and June 2019, patients with resectable esophageal cancer who underwent Ivor Lewis esophagectomy with a circular stapled anastomosis in the University Medical Center of the Johannes Gutenberg University (Mainz, Germany) were included in the analysis. Data on patient characteristics, surgical procedures, and postoperative outcomes were registered prospectively in an institutional database and were retrospectively analyzed. Postoperative complications were graded according to definitions stated by the modified Clavien-Dindo classification (MCDC) or the Esophagectomy Complications Consensus Group (ECCG).^15^,^16^ All anastomotic insufficiencies were symptomatic and were assessed by a CT scan with oral contrast and gastroscopy. All postoperative anastomotic strictures were symptomatic and were assessed by gastroscopy.

This study was approved by the medical ethical institutional review board of the Johannes Gutenberg University (JGU) of Mainz and the requirement to obtain informed consent was waived. Our department within the JGU is board certified and follows the guidelines of the German Cancer Society (DKG) for treatment of upper gastrointestinal cancer. Preoperative work-up included esophago-gastroscopy combined with endoscopic ultrasonography (EUS), tumor biopsy, and a combined thoracic and abdominal computed tomography (CT) scan. Positron emission tomography (PET)-CT scans were only used on indication when distant metastases outside the operative field were suspected. After preoperative work-up, patients were discussed in a multidisciplinary tumor board to determine optimal neoadjuvant and surgical treatment according to the current guidelines.

### Perioperative Management

All patients underwent a endoscopic preoperative dilatation of the pylorus on the day before esophagectomy to decrease the rate of delayed gastric emptying after Ivor Lewis esophagectomy.^17^

All patients received an epidural catheter to supply sufficient postoperative analgesia. Patients were intubated with a left-sided double-lumen tube to facilitate desufflation of the right lung. Prior to incision, intravenous antibiotic prophylaxis (sulbactam 1000 mg and ampicillin 2000 mg) was administered. No nasogastric tubes were placed postoperatively. Postoperatively, all patients were directly extubated in the operating room. Hereafter, patients were transferred to the intensive care unit (ICU) for respiratory and hemodynamic monitoring. On postoperative day 1, hemodynamically and respiratory stable patients were transferred to the surgical ward for further postoperative recovery. The first 3 days postoperatively, patients were placed on a nil-by-mouth routine. On the 4th day postoperatively, patients started with sips of water in absence of clinical signs of anastomotic insufficiency. Hereafter, oral intake was gradually increased to solid food. Esophageal swallow tests to prove or exclude anastomotic insufficiency were not routinely performed. There was no enhanced recovery or fast track program.

### Operating Procedure

There were 4 different approaches of Ivor Lewis esophagectomy with circular stapled intrathoracic anastomosis performed:

- Open transthoracic esophagectomy (OE)
- Hybrid esophagectomy (HE)
- Minimally invasive esophagectomy (MIE)
- Robot-assisted minimally invasive esophagectomy (RAMIE)

In OE, gastric mobilization and abdominal lymphadenectomy was performed through a laparotomy and esophageal mobilization with intrathoracic lymphadenectomy by a right-sided thoracotomy. HE was comparable to OE, but for the abdominal phase, a laparoscopic approach was used. In MIE, laparoscopic gastric mobilization and abdominal lymphadenectomy was followed by a minimally invasive thoracic phase. The RAMIE procedure was similar to MIE with the use of the Da Vinci® Xi robotic system (Intuitive Surgical Inc., Sunnyvale, CA, USA). In all procedures, the circular staple anvil (25 mm or 28 mm) was secured by a purse string suture. The circular stapled anastomosis was routinely oversewn and an omental wrap was placed around the anastomosis.^12^

All patients underwent esophagectomy with en bloc 2-field lymphadenectomy with Ivor Lewis gastric conduit reconstruction using a 25 mm or a 28 mm circular stapler (DST Series EEA, Medtronic, USA) for the esophagogastric anastomosis. The size of the circular stapler was chosen by the operating surgeon depending on the diameter of the lumen remaining proximal esophagus after transection in order to create an optimal esophagogastric anastomosis.

The esophagectomy resection specimen included a thoracic lymph node dissection with the paratracheal right-sided (lymph node station 2R), tracheobronchial (lymph node station 4), subcarinal (station 7), and peri-esophageal (station 8) lymph nodes.^18^,^19^ A D1+ abdominal lymphadenectomy was performed, including lymph nodes located at the portal vein, common hepatic artery, celiac trunk, left gastric artery, suprapancreatic, and lesser omental lymph nodes as well as lymph nodes around the splenic artery.^18^–^20^

### Pathological Analysis

The 8th edition of the Tumor Node Metastasis (TNM) classification stated by the International Union Against Cancer (UICC) was used to evaluate the resection specimen.^20^

The (circumferential) resection margins were examined using the criteria stated by the College of American Pathologists (CAP).^21^

### Statistical Analysis

Statistical analysis was performed using SPSS version 25.0 (SPSS, Chicago, IL, USA). All continuous data are presented as medians with range (minimum and maximum) or means with standard deviations. Results for categoric variables are presented as numbers with corresponding percentages. To evaluate significance of differences between groups, the Mann-Whitney *U* test was used for continuous variables and the chi-squared test was used as for categorical variables.

A binary univariate regression analysis was performed to identify risk factors for anastomotic insufficiency and postoperative anastomotic strictures. All outcomes with *p* < 0.10 in the univariate analysis and clinically relevant parameters were included in the binary multivariate regression analysis to identify independent risk factors for anastomotic insufficiency and postoperative anastomotic strictures.

## Results

Between February 2008 and June 2019, 349 patients with resectable esophageal cancer underwent Ivor Lewis esophagectomy with a circular stapled anastomosis. In 222 patients (64%), a 25 mm circular stapler was used, and in 127 (36%), a 28 mm device applied. There were no differences in baseline characteristics between the two groups and baseline characteristics were representative for patients with esophageal cancer in the Western world (Table 1).

**Table 1:** Patient demographics and tumor characteristics (*n* = 349)

	25 mm circular stapler (*n* = 222)	28 mm circular stapler (*n* = 127)	*p* value
Age (years) (median) (minimum–maximum)	63 (25–85)	64 (30–84)	0.328
Gender (*n* (%))			
Male	184 (83)	107 (85)	0.741
Female	38 (17)	20 (16)	
BMI (kg/m^2^) (median) (minimum–maximum)	26 (16–51)	25 (15–46)	0.210
Comorbidity (*n* (%))			
No comorbidity	47 (21)	37 (29)	0.094
Vascular	113 (51)	61 (48)	0.606
Cardiac	54 (24)	24 (18,9)	0.242
Diabetes	28 (13)	25 (20)	0.077
Pulmonary	50 (23)	20 (16)	0.128
Oncologic	21 (10)	10 (8)	0.616
Previous abdominal operation	71 (32)	33 (26)	0.239
Neurologic	15 (7)	10 (8)	0.570
ASA score (*n* (%))			
2	107 (48)	56 (44)	0.410
3	111 (50)	66 (52)	
4	4 (2)	5 (4)	
Clinical stage groups (TNM 8) (*n* (%))			0.313
cTxN0	8 (4)	1 (1)	
cTxN1	13 (6)	1 (1)	
cT1aN0	8 (4)	2 (2)	
cT1aN1	3 (1)	1 (1)	
cT1aN2	1 (1)	0 (0)	
cT1bN0	10 (5)	2 (2)	
cT1bN1	2 (1)	1 (1)	
cT1bN2	1 (1)	0 (0)	
cT2N0	24 (11)	13 (10)	
cT2N1	16 (7)	6 (5)	
cT2N2	2 (1)	1 (1)	
cT2N3	1 (1)	0 (0)	
cT3N0	23 (10)	18 (14)	
cT3N1	77 (35)	57 (45)	
cT3N2	18 (8)	11 (9)	
cT3N3	3 (1)	2 (2)	
cT4aN0	4 (2)	1 (1)	
cT4aN1	6 (3)	8 (6)	
cT4aN2	2 (1)	2 (2)	
Tumor location (*n* (%))			0.704
Upper esophageal	1 (1)	0 (0)	
Middle esophageal	25 (11)	16 (12)	
Lower esophageal/GEJ	196 (88)	111 (87)	
Tumor type (*n* (%))			0.217
Adenocarcinoma	165 (74)	97 (77)	
Squamous cell carcinoma	56 (25)	28 (22)	
Melanoma	1 (0)	0 (0)	
Neuro-endocrine	0 (0)	2 (2)	
Neoadjuvant treatment (*n* (%))			0.154
No therapy	66 (30)	25 (20)	
Chemotherapy	73 (33)	43 (34)	
Chemoradiotherapy	82 (37)	58 (46)	
Radiotherapy	1 (1)	0 (0)	

In the 25 mm group, there were significantly more OE and HE procedures, compared to the 28 mm group, where the majority of patients underwent RAMIE (*p* < 0.001). There were no differences in total operating time between the 25 mm and 28 mm groups (Table 2).

**Table 2:** Operative details (*n* = 349)

	25 mm circular stapler (*n* = 222)	28 mm circular stapler (*n* = 127)	*p* value
Operative approach			*p* < 0.001
Open	56 (25)	2 (2)	
Hybrid	81 (37)	14 (11)	
MIE	57 (26)	25 (20)	
RAMIE	28 (13)	86 (68)	
Operating time (min) (mean–SD)			
Total operating time	397 ± 79	384 ± 82	0.134

There were no differences in anastomotic insufficiency rates between the 25 mm (12%) and the 28 mm groups (11%) (*p* = 0.751). There were also no differences between postoperative anastomotic strictures in the 25 mm (14%) and the 28 mm groups (14%) (*p* = 0.863). Also, the median number of dilatations (*n* = 2 in each group, *p* = 0.573) and the time to first diagnosis of the stricture were not significantly different between the two groups (*p* = 0.412).

Considering further postoperative outcomes including pulmonary complications, cardiac complications, chylothorax, wound infections, and 30- and 90-day mortality, there were no differences between the 25 mm and 28 mm groups (Table 3).

**Table 3:** Postoperative data (*n* = 349)

	25 mm circular stapler (*n* = 222)	28 mm circular stapler (*n* = 127)	*p* value
Pulmonary complications (*n* (%))	84 (38)	36 (28)	0.072
Pneumonia	60 (27)	29 (23)	0.387
Cardiac complications (*n* (%))	35 (16)	18 (14)	0.690
Atrial fibrillation	32 (14)	18 (14)	0.951
Anastomotic insufficiency (*n* (%))	27 (12)	14 (11)	0.751
Anastomotic stricture	30 (14)	18 (14)	0.863
Number of dilatations (median) (minimum–maximum)	2 (0–19)	2 (0–5)	0.573
Time to stricture (days) (median) (minimum–maximum)	90 (27–938)	78 (12–243)	0.412
Chylothorax (*n* (%))	7 (3)	3 (2)	0.670
Recurrent laryngeal nerve palsy (*n* (%))	4 (2)	6 (5)	0.115
Wound infection (*n* (%))	26 (12)	9 (7)	0.166
30-day mortality	3 (1)	4 (3)	0.249
90-day mortality	13 (6)	6 (5)	0.654

A binary univariable and multivariable logistic regression analysis was performed to identify risk factors for anastomotic leakage (Table 4) after esophagectomy. The used stapler size (25 or 28 mm) was not associated with anastomotic leakage (HR 0.802 (95%CI, 0.345–1.862), *p* = 0.607) in univariate analysis. A medical history of diabetes was independently associated with the occurrence of anastomotic leakage in both uni- and multivariate analyses (HR 2.762 (95%CI 1.304–5.849), *p* = 0.008) (Table 4).

**Table 4:** Univariate and multivariate analysis of the association between risk factors and anastomotic leakage

Characteristic	Unadjusted HR (95%CI), univariate	*p*	Unadjusted HR (95%CI), multivariate	*p*
Stapler (25 versus 28 mm)	0.802 (0.345–1.862)	0.607		
No comorbidity	1.016 (0.429–2.405)	0.972		
Operative approach	1.012 (0.701–1.460)	0.950		
Diabetes	2.841 (1.273–6.338)	0.011	2.762 (1.304–5.849)	0.008

A binary univariable logistic regression analysis was performed to identify risk factors for postoperative anastomotic strictures (Table 5) after esophagectomy. The used stapler size (25 or 28 mm) was not associated with postoperative anastomotic strictures (HR 1.014 (95%CI 0.465–2.212, *p* = 0.972) in univariate analysis. Furthermore, anastomotic insufficiency was also not associated with the occurrence of postoperative anastomotic strictures (HR 0.457 (95%CI 0.134–1.559, *p* = 0.211) in univariate analysis (Table 5).

**Table 5:** Univariate and multivariate analysis of the association between risk factors and postoperative anastomotic strictures

Characteristic	Unadjusted HR (95%CI), univariate	*p*	Unadjusted HR (95%CI), multivariate	*p*
Stapler (25 versus 28 mm)	1.014 (0.465–2.212)	0.972		
No comorbidity	1.504 (0.680–3.326)	0.313		
Operative approach	1.063 (0.752–1.502)	0.729		
Diabetes	0.947 (0.383–2.339)	0.906		
Anastomotic leakage	0.457 (0.134–1.559)	0.211		

A radical resection (R0) was observed in 94% of patients in the 25 mm group and in 96% of patients in the 28 mm group (*p* = 0.436). There were no differences in postoperative pathological stage and histology between groups (Table 6).

**Table 6:** Histopathological data (*n* = 349)

	25 mm circular stapler (*n* = 222)	28 mm circular stapler (*n* = 127)	*p* value
Histological type (*n* (%))			0.360
Adenocarcinoma	151 (68)	88 (69)	
Squamous cell carcinoma	44 (20)	22 (17)	
Melanoma	1 (1)	0 (0)	
Neuro-endocrine	0 (0)	2 (2)	
No viable tumor cells	26 (12)	15 (12)	
Radicality(*n* (%))			0.436
R0	209 (94)	122 (96)	
R1	13 (6)	5 (4)	
Pathological stage groups (TNM 8) (*n* (%))			0.110
pT0N0	26 (12)	15 (12)	
pT0N1	5 (2)	2 (2)	
pT0N2	2 (1)	0 (0)	
pT1aN0	18 (8)	5 (4)	
pT1aN2	2 (1)	0 (0)	
pT1bN0	31 (14)	8 (6)	
pT1bN1	7 (3)	1 (1)	
pT1bN2	3 (1)	2 (2)	
pT1bN3	0 (0)	2 (2)	
pT2N0	19 (9)	13 (10)	
pT2N1	16 (7)	5 (4)	
pT2N2	7 (3)	2 (2)	
pT2N3	3 (1)	1 (1)	
pT3N0	32 (14)	22 (17)	
pT3N1	13 (6)	12 (9)	
pT3N2	19 (9)	19 (15)	
pT3N3	16 (7)	15 (12)	
pT4aN0	0 (0)	1 (1)	
pT4aN1	1 (1)	0 (0)	
pT4aN2	1 (1)	0 (0)	
pT4aN3	1 (1)	2 (2)	

## Discussion

In this study, the 25 mm circular stapler was compared to the 28 mm stapler for a circular stapled esophagogastric anastomosis after Ivor Lewis esophagectomy. There were no differences between the 25 and 28 mm circular stapler considering anastomotic insufficiency, postoperative anastomotic strictures, the number of dilations needed, and the time to the first diagnosis of postoperative anastomotic stricture. These data clearly show that both the 25 mm and the 28 mm stapler can be used safely to create the intrathoracic esophagogastric anastomosis to restore continuity after esophagectomy. Furthermore, in this study, the only finding which was independently associated with the occurrence of anastomotic insufficiency was a medical history of diabetes.

Currently, there is only one meta-analysis, which compared different circular stapler diameters (25 mm versus 28 mm) to create the esophagogastric anastomosis.^22^ In this meta-analysis, 5 observational studies were included.^23^–^28^ It was concluded that the use of larger circular stapler size (28 mm versus 25 mm) was strongly associated with a reduced risk of postoperative anastomotic strictures in the upper GI tract.^22^ Results from our study do not support the conclusion of aforementioned meta-analysis, as there were no differences observed in anastomotic insufficiency and postoperative anastomotic strictures between the 25 mm and the 28 mm stapled esophagogastric anastomosis. The meta-analysis included 367 patients in the 25 mm and 460 patients in the 28 mm group (827 patients in total).^22^ Our study included 349 patients (222 in the 25 mm group and 127 in the 28 mm group) and might significantly contribute (30% additional patients) evidence that there is no difference in postoperative outcomes between the two stapler diameters applied for a circular stapled intrathoracic anastomosis.

In our study, both postoperative anastomotic stricture rates after the 25 mm (14%) and the 28 mm stapled anastomosis (14%) were comparable to the rate reported (28 mm, 11%) in the largest study included in the aforementioned meta-analysis.^24^ This shows that our postoperative anastomotic stricture rates observed in our cohort were comparable to other high volume tertiary referral centers and reflect the incidence of postoperative anastomotic stricture rates in daily practice.^22^,^24^ The size of the circular stapler (25 mm or 28 mm) as used in this study was chosen based on the size of the esophageal lumen after transection. Without a difference in the incidence of anastomotic insufficiency and postoperative anastomotic dilatations after esophagectomy, the surgeon could choose either the 25 mm or 28 mm circular stapler to restore continuity based on the estimation of the esophageal lumen. The question, which is the best surgical strategy to create the “ideal” esophagogastric anastomosis after esophagectomy for distal esophageal cancer or cancer of the gastro-esophageal junction, still remains. Results from a meta-analysis including observational studies comparing an Ivor Lewis esophagectomy to a McKeown esophagectomy showed that an Ivor Lewis esophagectomy was associated with a lower incidence of anastomotic leakage, 90-day mortality, and other postoperative morbidity.^6^ However, randomized evidence is yet still lacking. The ICAN randomized controlled trial will provide evidence, whether the McKeown procedure is associated with a higher percentage of postoperative complications compared to the Ivor Lewis procedure.^7^ With regard to the latter, it is still unclear how to perform the best anastomotic technique.

In the 25 mm group, more than 50% of patients had either an open or hybrid esophagectomy, both through a right thoracotomy. However, in our minimally invasive group, mainly a right mini-thoracotomy was used with a 28 mm stapler. This could raise questions about the technical feasibility, quality, and performance of the intrathoracic esophagogastric anastomosis. In one of our previous publications, we showed that restoring continuity through a right mini-thoracotomy did not compromise the quality and performance of the anastomosis.^29^

Limitations of this study include the single-center design and a time bias for the 25 mm anastomosis, according to the surgeon’s preference, not the measured diameter of the esophageal lumen. Furthermore, there were no postoperative quality of life and nutritional questionnaires obtained within this study. Results of these questionnaires might show important postoperative functional results of the 25 mm and 28 mm stapled anastomosis technique. However, these—in general—have to be interpreted with caution, as they are based on patients’ subjective assessment and, thus, not of major importance with regard to the objectives of our current study.

In this study, there were no differences in anastomotic insufficiency and postoperative anastomotic strictures between a 25 mm and 28 mm circular stapled gastro-esophageal anastomosis after Ivor Lewis esophagectomy. Randomized controlled trials are needed in order to answer the question, which is the best technique to perform the “ideal” esophagogastric anastomosis after Ivor Lewis esophagectomy.

## Data Availability

The datasets used and analyzed during the current study are anonymously available from the corresponding author on reasonable request.
